# Supplementary figures and images for: Analysis of the Humoral Immunal Response Transcriptome of Ectropis obliqua Infected by Beauveria bassiana
Source: Insects. 2022 Feb 24;13(3):225. doi: 10.3390/insects13030225 (PMC8955196; doi:10.3390/insects13030225)

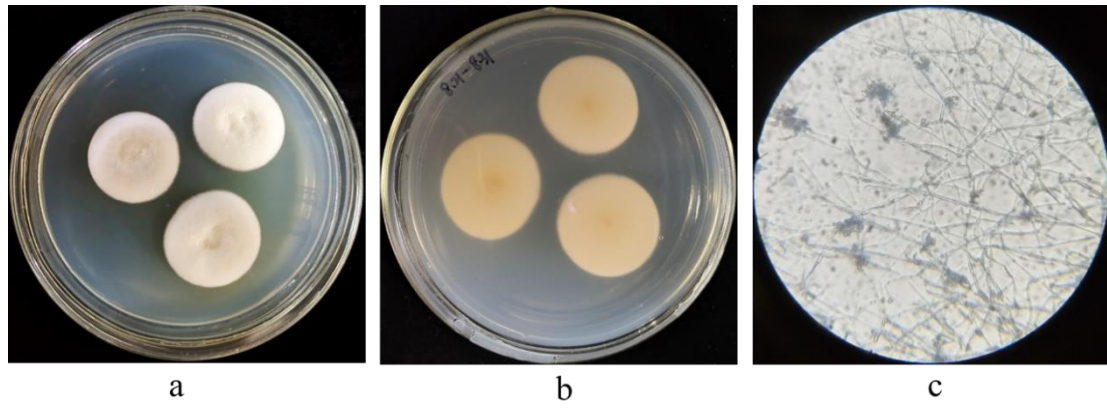

Figure S1. Morphological characteristics of strain and optical microscopic observation

Supplement: Supplementary file 1 [file insects-13-00225-s001.zip › Figure S1. Morphological characteristics of strain and optical microscopic observation.pdf]
